# Comparison between ultra-high and conventional mono b-value DWI for preoperative glioma grading

**DOI:** 10.18632/oncotarget.14180

**Published:** 2016-12-26

**Authors:** Yu-Chuan Hu, Lin-Feng Yan, Qian Sun, Zhi-Cheng Liu, Shu-Mei Wang, Yu Han, Qiang Tian, Ying-Zhi Sun, Dan-Dan Zheng, Wen Wang, Guang-Bin Cui

**Affiliations:** ^1^ Department of Radiology, Tangdu Hospital, Fourth Military Medical University, Xi’an, China; ^2^ Department of Pathology, Tangdu Hospital, Fourth Military Medical University, Xi’an, China; ^3^ MR Research China, GE Healthcare China, Beijing, China

**Keywords:** glioma, grading, diffusion-weighted imaging, MRI, apparent diffusion coefficient

## Abstract

To compare the efficacy of ultra-high and conventional mono-b-value DWI for glioma grading, in 109 pathologically confirmed glioma patients, ultra-high apparent diffusion coefficient (ADC_uh_)was calculated using a tri-exponential mode, distributed diffusion coefficients (DDCs) and α values were calculated using a stretched-exponential model, and conventional ADC values were calculated using a mono-exponential model. The efficacy and reliability of parameters for grading gliomas were investigated using receiver operating characteristic (ROC) curve and intra-class correlation (ICC) analyses, respectively. The ADC_uh_ values differed (P < 0.001) between low-grade gliomas (LGGs; 0.436 ×10^−3^ mm^2^/sec) and high-grade gliomas (HGGs; 0.285 × 10^−3^ mm^2^/sec). DDC, a and various conventional ADC values were smaller in HGGs (all P ≤ 0.001, vs. LGGs). The ADC_uh_ parameter achieved the highest diagnostic efficacy with an area under curve (AUC) of 0.993, 92.9% sensitivity and 98.8% specificity for glioma grading at a cutoff value of 0.362×10^−3^ mm^2^/sec. ADC_uh_ measurement appears to be an easy-to-perform technique with good reproducibility (ICC = 0.9391, P < 0.001). The ADC_uh_ value based in a tri-exponential model exhibited greater efficacy and reliability than other DWI parameters, making it a promising technique for glioma grading.

## INTRODUCTION

The preoperative grading of gliomas, which is critical to determine the optimal therapy, remains unsatisfactory [1, 2]. Histopathology remains the gold standard for brain glioma diagnosis [3]. However, the histopathological grading of glioma is frequently biased because of the intratumoral heterogeneity of the tumor sample from stereotactic biopsy or surgical resection. This biased histopathological grading leads to the improper therapeutic strategy [4]. An unbiased preoperative grading based on the information of the whole tumor is urgently needed. Advanced MRI methods fit well into this frame in that they permit more uniform sampling of the whole tumor than obtained by heterogeneous biopsies, and thus demonstrate a promising future in glioma grading.

Diffusion-weighted imaging (DWI) is considered to be the most sensitive to detect early pathological changes and demonstrated potentials for noninvasive glioma grading in previous studies [3, 5, 6]. A series of diffusion-weighted models using an extended b-value range have been introduced to describe the different aspects of tissue diffusion properties [7]. Conventional DWI based on only two b-values (so-called mono-exponential model, usually 0 and 1000 sec/mm^2^ in the brain) provides unique information on tissue functional structure [8]. Intravoxel incoherent motion (IVIM) DWI can be used to extract the perfusion-related information from a diffusion sequence by collecting both low b-values (< 200 sec/mm^2^) and high b-values (usually 200 - 1000 sec/mm^2^) *in vivo* [9], therefore providing information on tumor cellularity and microcirculation without using contrast. Unlike conventional DWI, the stretched exponential model provides a new type of image contrast, reflecting the extent of intravoxel water diffusion heterogeneity [10].

Recently, ADC values derived from the high b-values (b = 3000 sec/mm^2^) DWI were reported to improve the diagnostic performance in differentiating high- from low-grade gliomas [11]. However, a conventional mono-exponential model was used in that study. Furthermore, ADC_uh_ (calculated by fitting the signals at ultra-high b-values: 2,000 – 5,000 sec/mm^2^) values of the globus pallidus, putamen, and substantia nigra were significantly lower in Parkinson's disease (PD) patients than those in control subjects, while standard ADC (ADC_st_, calculated by fitting the standard b-values: 0, 1,000 sec/mm^2^), pure diffusion coefficient (D) and pseudo-diffusion coefficient (D*) values from the corresponding regions of PD patients were not significantly changed [12]. There is also evidence that ADC may be related to the membrane expression of aquaporin-4 (AQP4) [13–15]. AQP expression of gliomas correlate with tumor type, grade, proliferation, angiogenesis, cell migration and invasion [16–19]. We therefore hypothesize that ADC_uh_ is useful in grading gliomas.

It is generally accepted that the various DWI models have varied potential for preoperative glioma grading [2–4, 20]. It is therefore important to compare the diagnostic efficacy and reliability of different DWI parameters so as to design optimal scan protocols. We retrospectively compared the efficacy and reliability among tri-, mono- and stretched-exponential model DWI for glioma grading, and a tri-component model was used to calculate ADC_uh_ value based on 18 b-values (up to 4,500 sec/mm^2^).

## RESULTS

### Baseline characteristics

The clinical and demographic characteristics of the 109 glioma patients are summarized in Table 1. The study group consisted of 70 males and 39 females with a mean age of 46.9 ± 17.2 years (range: 2 – 87 years). Among these patients, 28 patients were pathologically diagnosed as LGG (WHO grade I: pilocytic astrocytoma (n = 3); Grade II: diffuse astrocytoma (n = 6), oligodendroglioma (n = 3), oligoastrocytoma (n = 13), ependymoma (n = 2) and pleomorphic xanthoastrocytoma (n = 1)), and 81 as HGG (WHO grade III: anaplastic astrocytoma (n = 7), anaplastic oligoastrocytoma (n = 14) and anaplastic oligodendroglioma (n = 3) ; Grade IV: glioblastoma (n = 57)). The clinical presentations of the patients were headache and vomiting (35.8%; 43 of 109 patients), dizziness (20.8%; 20 of 109), epilepsy (14.2%; 17 of 109), physical dysfunction (7.5%; 9 of 109) and others (10.0%; 12 of 109), as well as no apparent symptoms (2.5%).

**Table 1:** Baseline characteristics of the 109 glioma patients

Patient characteristic	
**Age (yrs)**	
Mean ± SD	46.9 ± 17.2
Median	50.0
Range	2-87
**Sex - no.(%)**	
Males	70 (58.3)
Females	39 (32.5)
**Major symptoms or signs - no.(%)**	
No symptom	3 (2.5)
Dizziness	25 (20.8)
Epilepsy	17 (14.2)
Headache, vomiting	43 (35.8)
Physical dysfunction	9 (7.5)
Others	12 (10.0)
**Method for obtaining pathologic results - no.(%)**	
Surgery	109 (100.0)
Puncture biopsy	0 (0)
**Pathological types - no.(%)**	
**LGG**	28 (25.7)
Pilocytic astrocytoma, grade I	3 (2.8)
Diffuse astrocytoma, grade II	6 (5.5)
Oligodendroglioma, grade II	3 (2.8)
Oligoastrocytoma, grade II	13 (11.9)
Ependymoma, grade II	2 (1.8)
Pleomorphic xanthoastrocytoma, grade II	1 (0.9)
**HGG**	81 (74.3)
Anaplastic astrocytoma, grade III	7 (6.4)
Anaplastic oligoastrocytoma, grade III	14 (12.8)
Anaplastic oligodendroglioma, grade III	3 (2.8)
Glioblastoma, grade IV	57 (52.3)

Note: LGG = low-grade glioma; HGG = high-grade glioma.

### Parametics comparison between LGG and HGG

**Figure 1:**
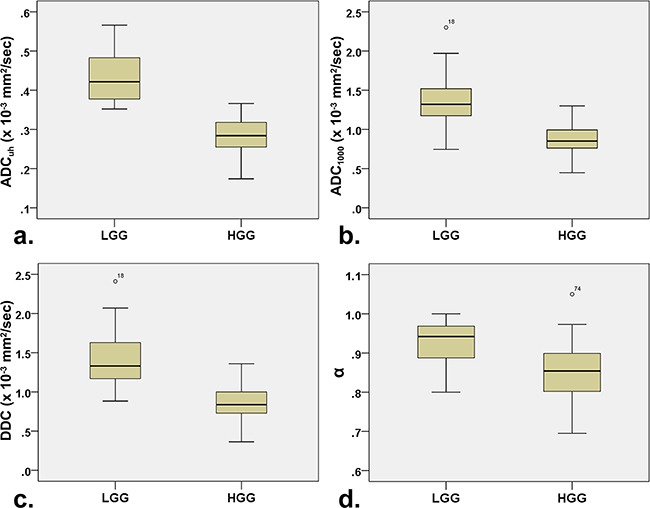
Box plots for values a ADC_uh_, **b**. ADC_1000_, **c**. DDC and **d**. α between low- and high-grade gliomas. Note: LGG = low-grade gliomas; HGG = high-grade gliomas; ADC_uh_ = ADC calculated using the ultra-high b-values from tri-component model DWI; DDC = distributed diffusion coefficient; α = water molecular diffusion heterogeneity index; ADC_1000_ = ADC calculated using mono-exponential model DWI (b value = 0,1000 sec/mm^2^).

**Figure 2:**
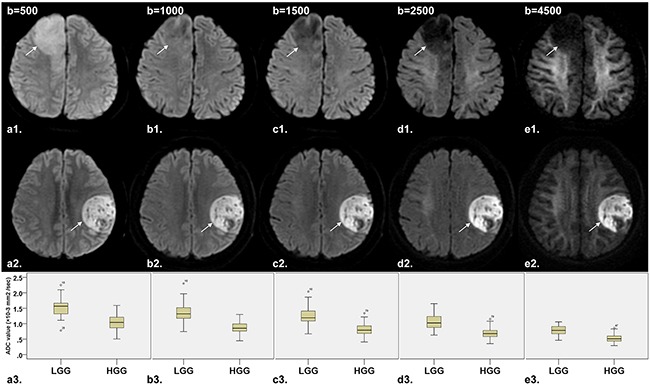
Comparison of DW images (b=500, 1000, 1500, 2500 and 4500 sec/mm^2^, respectively) and corresponding box plot between LGG (diffuse astrocytoma in right frontal lobe, *arrow*) and HGG (glioblastoma in left frontal lobe, *arrow*) **a1-e1**. Axial DW image shows that the signal of tumor tissue in LGG decreases rapidly with increasing diffusion weighting. **a2-e2**. Axial DW image shows no significant decreased signal of tumor tissue in HGG with increasing diffusion weighting. **a3-e3**. Box plot of ADC value between LGG and HGG shows that ADC value decreased along with the increase of b value both in LGG and HGG, with significant difference of ADC value between LGG and HGG. Note: LGG = low-grade gliomas; HGG = high-grade gliomas.

The descriptive statistics of the DWI parameters comparison between LGG and HGG is shown in Table 2. The mean ADC_uh_ value was 0.436 × 10^−3^ mm^2^/sec in LGG and 0.285 × 10^−3^ mm^2^ /sec in HGG with significant differences (P < 0.001) (Figure 1a), while no significant differences were found in peritumoral edema area (ADC_uh_edema_) and contralateral healthy white matter area (ADC_uh_wm_) (P > 0.05). For the parameters from the stretched-exponential model, DDC and α values were significantly decreased in HGG patients (0.869 × 10^−3^ vs. 1.427 × 10^−3^ mm^2^/sec, P < 0.001; 0.853 vs. 0.928, P < 0.001, respectively) (Figure 1c and 1d). Significant differences between LGG and HGG were also detected for all twelve conventional ADC values (b = 500 - 4,500 sec/mm^2^) (Figure 1b and Figure 2a3-e3), with significantly higher mean ADC value in the LGG patients (P < 0.001). With increasing diffusion weighting, the signal of the tumor tissue in LGG decreases more rapidly (Figure 2a1-e1) than the signal in HGG (Figure 2a2-e2).

**Table 2:** ADC values comparison between tri-component model and conventional mono-b value DWI in low- and high-grade gliomas (x¯ ± *s*)

Models	Values	LGG	HGG	t	P-value
**Tri-exponential model**	ADC_uh_ (×10^−3^ mm^2^/sec)	0.436 ± 0.062	0.285 ± 0.045	11.847	< 0.001
	ADC_uh_edema_(×10^−3^ mm^2^/sec)	0.273 ± 0.060	0.262 ± 0.053	.794	0.429
	ADC_uh_wm_ (×10^−3^ mm^2^/sec)	0.213 ± 0.025	0.206 ± 0.024	1.365	0.175
**Stretched-exponential Model**	DDC(10^-3^mm^2^/sec)	1.427 ± 0.394	0.869 ± 0.212	7.137	< 0.001
	α	0.928 ± 0.053	0.853 ± 0.068	5.312	< 0.001
**Mono-exponential Model**	ADC_500_(×10^−3^ mm^2^/sec)	1.533 ± 0.319	1.057 ± 0.254	7.977	< 0.001
	ADC_800_(×10^−3^ mm^2^/sec)	1.445 ± 0.332	0.959 ± 0.218	8.798	< 0.001
	ADC_1000_(×10^−3^ mm^2^/sec)	1.364 ± 0.334	0.895 ± 0.192	7.037	< 0.001
	ADC_1300_(×10^−3^ mm^2^/sec)	1.301 ± 0.323	0.850 ± 0.193	6.964	< 0.001
	ADC_1500_(×10^−3^ mm^2^/sec)	1.254 ± 0.324	0.816 ± 0.187	6.770	< 0.001
	ADC_1700_(×10^−3^ mm^2^/sec)	1.203 ± 0.323	0.785 ± 0.179	6.493	< 0.001
	ADC_2000_(×10^−3^ mm^2^/sec)	1.149 ± 0.303	0.741 ± 0.168	6.772	< 0.001
	ADC_2500_(×10^−3^ mm^2^/sec)	1.067 ± 0.277	0.691 ± 0.156	6.813	< 0.001
	ADC_3000_(×10^−3^ mm^2^/sec)	0.982 ± 0.230	0.647 ± 0.148	7.220	< 0.001
	ADC_3500_(×10^−3^ mm^2^/sec)	0.914 ± 0.209	0.599 ± 0.139	7.418	< 0.001
	ADC_4000_(×10^−3^ mm^2^/sec)	0.845 ± 0.180	0.563 ± 0.133	8.791	< 0.001
	ADC_4500_(×10^−3^ mm^2^/sec)	0.780 ± 0.154	0.531 ± 0.124	8.582	< 0.001

Note: *The P values were the comparison of the parameters between LGG and HGG. LGG = low-grade gliomas; HGG = high-grade gliomas; ADC_uh_ = ADC calculated using the ultra-high b-values from tri-component model DWI; DDC = distributed diffusion coefficient; α = water molecular diffusion heterogeneity index; ADC_n_ = ADC calculated using mono-exponential model DWI (b value= 0, n sec/mm^2^).

### Diagnostic efficacy of ADC_uh_ was higher than other parameters

**Figure 3:**
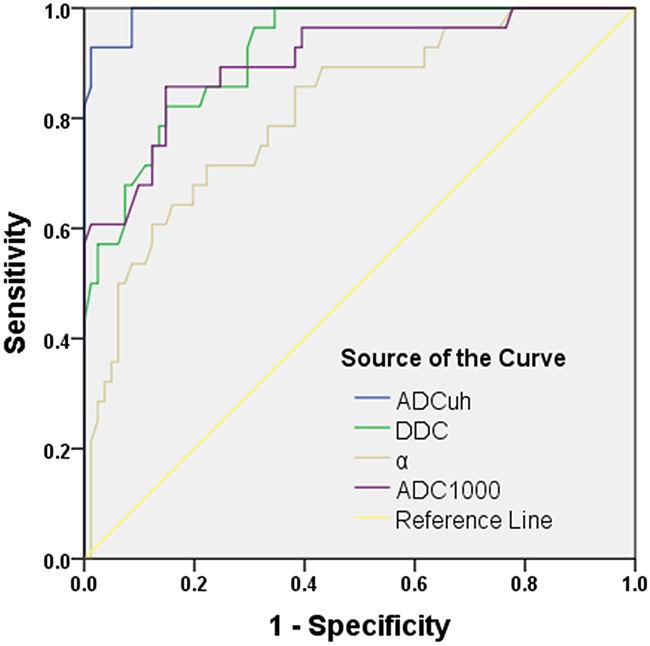
ROC curve for the differentiating performances of the ADC_uh_, DDC, α and ADC_1000_ value between low- and high-grade gliomas Note: ADC_uh_ = ADC calculated using the ultra-high b-values from tri-component model DWI; ADC_1000_ = ADC calculated using mono-exponential model DWI (b value= 0,1000 sec/mm^2^); DDC = distributed diffusion coefficient; α = water molecular diffusion heterogeneity index.

According to the ROC analyses, the ADC_uh_ parameter achieved a highest diagnostic efficacy of sensitivity (92.9%) and specificity (98.8%) with AUC of 0.993 at the cutoff value of 0.362 × 10^−3^ mm^2^/sec (Table 3 and Figure 3).

**Table 3:** ROC curve

Values	AUC	P value	Sensitivity(%)	Specificity(%)	Cutoff value
ADC_uh_ (×10^−3^ mm^2^/sec)	0.993	< 0.001	92.9	98.8	0.362
DDC (10^-3^ mm^2^/sec)	0.920	< 0.001	82.1	85.2	1.125
α	0.814	< 0.001	71.4	77.8	0.908
ADC_500_(×10^−3^ mm^2^/sec)	0.884	< 0.001	85.7	77.8	1.225
ADC_800_(×10^−3^ mm^2^/sec)	0.904	< 0.001	85.7	81.5	1.175
ADC_1000_(×10^−3^ mm^2^/sec)	0.905	< 0.001	82.7	85.2	1.115
ADC_1300_(×10^−3^ mm^2^/sec)	0.896	< 0.001	85.7	85.2	1.030
ADC_1500_(×10^−3^ mm^2^/sec)	0.892	< 0.001	78.6	90.1	1.075
ADC_1700_(×10^−3^ mm^2^/sec)	0.888	< 0.001	78.6	87.7	0.984
ADC_2000_(×10^−3^ mm^2^/sec)	0.901	< 0.001	82.1	90.1	0.946
ADC_2500_(×10^−3^ mm^2^/sec)	0.898	< 0.001	75.0	90.1	0.906
ADC_3000_(×10^−3^ mm^2^/sec)	0.897	< 0.001	85.7	81.5	0.763
ADC_3500_(×10^−3^ mm^2^/sec)	0.897	< 0.001	82.1	87.7	0.757
ADC_4000_(×10^−3^ mm^2^/sec)	0.895	< 0.001	82.1	86.4	0.726
ADC_4500_(×10^−3^ mm^2^/sec)	0.890	< 0.001	82.1	82.7	0.656

Note: AUC = area under curve; ADC_uh_ = ADC calculated using the ultra-high b-values from tri-component model DWI; DDC = distributed diffusion coefficient; α = water molecular diffusion heterogeneity index; ADC_n_= ADC calculated using mono-exponential model DWI (b value = 0, n sec/mm^2^).

The AUC, sensitivity, specificity and the cutoff value, respectively, for differentiating LGG from HGG for DDC and α (Table 3 and Figure 3) were as follows: DDC, 0.920, 82.1%, 85.2% and 1.125 × 10^−3^ mm^2^/sec; α, 0.814, 71.4%, 77.8% and 0.908.

For each parameter from conventional mono-exponential model DWI (b-value: 500, 800, 1000, 1300, 1500, 1700, 2000, 2500, 3000, 3500, 4000 and 4500 sec/mm^2^), the values of AUC, sensitivity, specificity, as well as the suggestive cutoff values are shown in Table 3. Briefly, ADC_1000_ parameter achieved a relatively higher diagnostic efficacy with AUC of 0.905, 82.7% sensitivity and 85.2% specificity for differentiating the LGG at the cutoff value of 1.115 × 10^−3^ mm^2^/sec (Figure 3), with a similar diagnostic efficacy for other ADC values from mono-exponential model.

### Data reliability analysis

As shown in Table 4, the inter-observer ICC value for ADC_uh_ was close to 1 (P < 0.001), and those for DDC and ADC_st_ were all higher than 0.75 (all P < 0.001), suggesting very good measurement reliability of quantitative MRI parameters. However, relatively poor measuring consistencies in α (0.719) were revealed.

**Table 4:** Reliability analysis between the first and second measuring of the parameters

Values	ICC	P value	95% CI
ADC_uh_ (×10^−3^ mm^2^/sec)	0.939	< 0.001	0.912 - 0.958
DDC (10^-3^ mm^2^/sec)	0.904	< 0.001	0.863 - 0.934
α	0.719	< 0.001	0.617 - 0.799
ADC_1000_ (×10^−3^ mm^2^/sec)	0.838	< 0.001	0.771 - 0.886

Note: ICC = intraclass correlation; 95% CI = 95% confidence interval; ADC_uh_ = ADC calculated using the ultra-high b-values from tri-component model DWI; DDC = distributed diffusion coefficient; α = water molecular diffusion heterogeneity index; ADC_1000_ = ADC calculated using mono-exponential model DWI (b value = 0,1000 sec/mm^2^).

## DISCUSSION

In the current study, we identified that the ADC_uh_ value based on tri-exponential model DWI was useful in glioma grading and demonstrated a higher efficacy and reliability over other parameters from DWI models. We also determined the most appropriate cutoff values for different DWI parameters, which could potentially be used in clinical practice regarding preoperative glioma grading.

Previous studies suggest that ADC and D values were significantly lower in HGG than in LGG patients, owing to the increased cellularity and nuclear cytoplasmic ratio [2, 4, 11, 20, 21]. Similarly, we detected significantly decreased ADC_uh_ in HGG than LGG (P < 0.001). Interestingly, we noticed that ADC_uh_ was dramatically lower than the conventional ADC values, even the ADC_4500_ both in LGG and HGG. Two possible explanations for this difference are as follows. First, according to the mono exponential model, the diffusion signal intensity and ADC value of tumor constantly decreased with the increasing b-value [3, 7]. Second, there is growing evidence that AQP in the membranes is the main inhibitor of water diffusion [12]. For example, AQP4 is the key molecule involved in brain water homeostasis [22, 23], and can modulate ADC values under normal and pathological conditions [24, 25]. Therefore, the significant differences of ADC_uh_ between low- and high-grade gliomas may indicate the different levels of AQP expression.

In our study, we also evaluated glioma grading using multiple b-value DWI based on the stretched exponential model, and we found that the DDC and α value of HGG were significantly lower than the low grade ones. This finding was consistent with the previous studies [10, 20], and can be explained by the fact that HGG, in particular glioblastoma (52.3% of included patients), is associated with considerable histological heterogeneity [10, 20, 26]. DDC and α value demonstrated a relative good differentiating diagnostic efficacy, and the stretched exponential model may play a potential role in glioma grading.

Although using a greater number of b-values can improve the accuracy of the ADC measurements [27], the conventional mono-exponential model has been successfully and widely used today in clinical practice. In our study, we confirmed that conventional ADC values from different b-values were valuable in glioma grading, but the ADC_1000_ achieved the highest differentiating diagnostic efficacy among all the other ADC values.

We noticed that sometimes it may be difficult to recognize the anaplastic components (grade III) under the background of grade II gliomas, at the relative lower b-value (b-value ≤ 1000 sec/mm^2^) DW images, such as b-value of 500 or 800 sec/mm^2^, while it may be clear delineated on the more high b-value (b-value ≥ 1500 sec/mm^2^) DW images. Similar results were indicated in a previous study, in which it was shown that T2-weighted images plus DW images with a b-value of 2,000 sec/mm^2^ are superior to T2-weighted images plus DW images with a b-value of 1,000 sec/mm^2^ in the detection of prostate cancer [28]. In addition, the valuable quantitative information on biological tissue may be missed because of the fact that low b-values of a few hundreds to 1000 sec/mm^2^ usually reflect the extracellular space, and the complex intracellular space and membrane interactions remain invisible [7]. The present study showed a striking diffusion signal decay in LGG, with no significant signal decay in HGG on the DW images at ultra high b-value (b-value ≥ 2000 sec/mm^2^), which can be used to the glioma grading visually.

In this study, the ICC analysis was performed to explore the measurement consistency between the two independent radiologists. The results showed that ICC value of ADC_uh_ was close to 1, and ICC value of DDC and ADC_1000_ were all higher than 0.75. However, the ICC value of α was relative low. To ensure data accuracy, we also obtained ADC_uh_ data from contralateral healthy white matter area and peritumoral edema area in glioma patients. Analysis revealed no significant difference for ADC_uh_edema_ and ADC_uh_wm_ between low- and high-grade gliomas, which also suggested the reliability of the ADC_uh_ data in our study.

### Limitations

Our study has some limitations. First, as an initial research on tri-exponential model based on MR DWI, we didn't analyze the correlation between the levels of AQP expression and ADC_uh_ in gliomas, and further research is warranted to clarify this issue. Second, hand-drawn ROIs were used in the current study. Since the nests of tumor cells tend to be heterogeneously distributed, a measurement of ADC values by manual defining ROIs may lead to sampling bias. Finally, we didn't compare the IVIM parameters because of the fact that we only used four b-values within 200 sec/mm^2^ for a bi-exponential model, which could be insufficient to measure perfusion-related parameters accurately.

## MATERIALS AND METHODS

### Subjects

**Figure 4:**
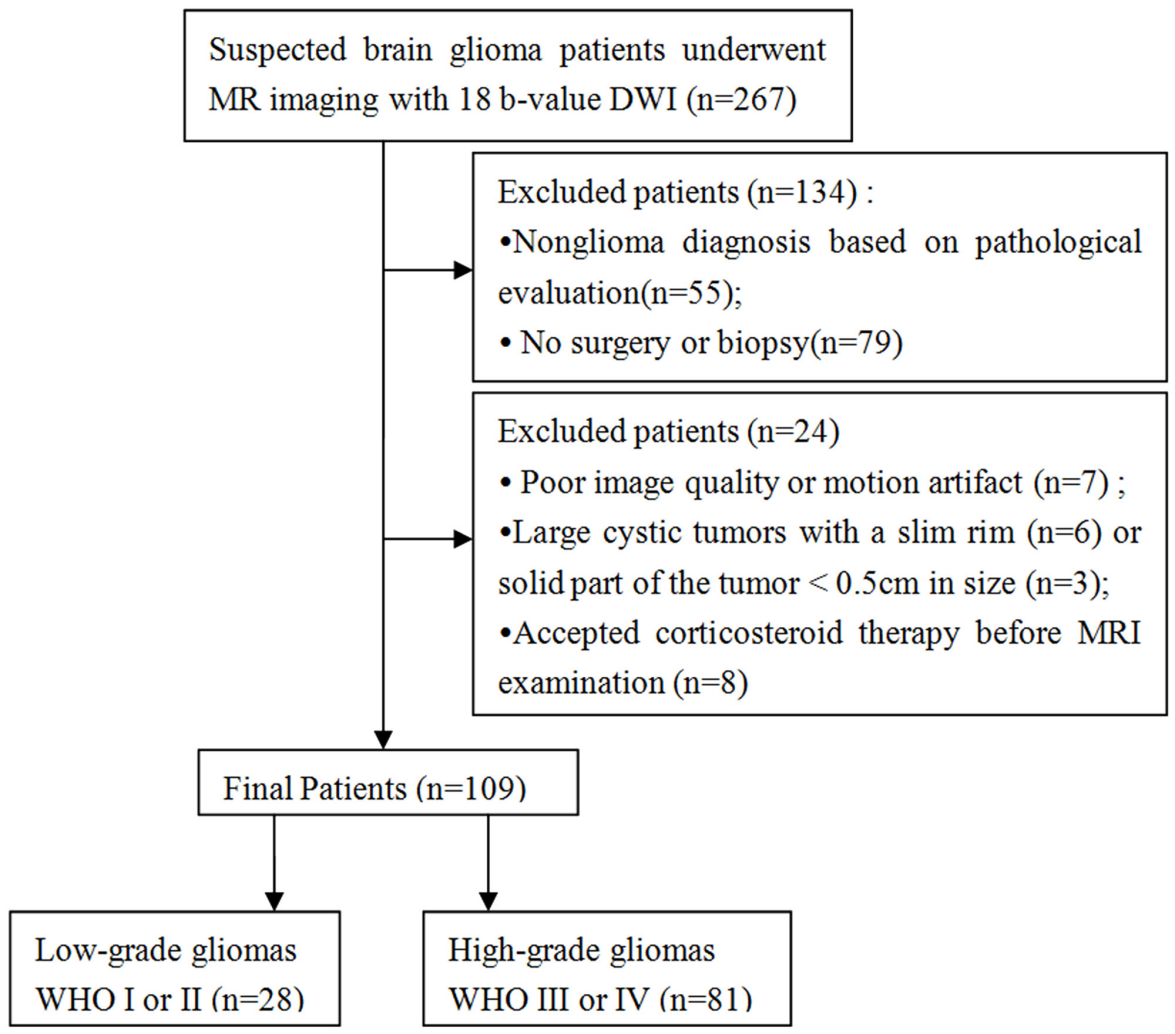
Flow diagram of patient selection and inclusion and exclusion criteria

Between July 2014 and September 2015, 267 patients with suspected glioma underwent routine MRI, 18 b-value DWI, as well as contrast-enhanced MRI of the brain before any treatment. Among them, 55 patients were excluded for pathologically confirmed nonglioma; 79 patients were excluded for no pathological diagnosis due to the absence of surgical or biopsy samples; and 24 patients were excluded for the following reasons: poor image quality or motion artifact (n = 7); large cystic tumors with a slim rim (n = 6) or solid tumor is < 0.5 cm in diameter (n = 3); and receiving corticosteroid therapy before MRI examination (n = 8). Finally, 109 pathologically confirmed glioma patients (mean age, 50 years; range, 2 - 87 years) were enrolled in the current study (Figure 4 and Table 1). This retrospective study was approved by the local ethics committee, and informed consent was waived.

### Brain MR imaging

The whole brain MRI examinations were performed on a 3.0-T MRI system (MR750, GE Healthcare, Milwaukee, WI, USA) with a standard receive-only head coil. Conventional MRI, DWI with 18 b-values (0–4500 sec/mm^2^) and contrast-enhanced MRI were performed during the same examination.

Conventional MRI sequences included: T1-weighted spin echo in the transverse plane (TR/TE, 1,750 ms/24 ms; matrix size, 256 × 256; field of view (FOV), 24 cm × 24 cm; number of excitation (NEX), 1; slice thickness, 5 mm; gap, 1.5 mm); T2-weighted fast spin echo in the transverse planes (TR/TE, 4,247 ms/93 ms; matrix size, 512 × 512; FOV, 24 cm × 24 cm; NEX, 1; slice thickness, 5 mm; gap, 1.5 mm) and sagittal planes (TR/TE, 10,639 ms/96 ms; matrix size, 384 × 384; FOV, 24 cm × 24 cm; NEX, 2; slice thickness, 5 mm; gap, 1.0 mm); and fluid-attenuated inversion recovery (FLAIR) in the transverse plane (TR/TE, 8,000 ms/165 ms; matrix size, 256 × 256; FOV, 24 cm × 24 cm; NEX, 1; slice thickness, 5 mm; gap, 1.5 mm).

The multi-b-value DWI sequence was performed prior to the contrast injection. Eighteen b-values (0, 50, 100, 150, 200, 300, 500, 800, 1000, 1300, 1500, 1700, 2000, 2500, 3000, 3500, 4000 and 4500 sec/mm^2^) were applied with a single-shot diffusion-weighted spin-echo echo-planar sequence. The lookup table of gradient directions was modified to allow multiple b-value measurements in one series. Parallel imaging was used with an acceleration factor of 2. A local shim box covering the whole brain was applied to minimize susceptibility artifacts. In total, 20 axial slices covering the entire brain were obtained with a 24 cm × 24 cm FOV, 5 mm slice thickness, 1.5 mm slice gap, 3,000 ms TR, Minimum TE, 128 × 128 matrix. With the increase of b-values, the NEX also increased from one to five to ensure a good signal noise ratio. The total scan time was 5 min and 31 sec.

Finally, a contrast-enhanced T1-weighted spin echo sequence was performed in the transverse, sagittal, and coronal planes (TR/TE, 1850 ms/24 ms for transverse plane and 1750 ms/24 ms for sagittal and coronal planes; other parameters were the same as conventional MRI) following a bolus injection of 0.1 mmol/kg of gadodiamide (Omniscan; GE Healthcare, Co.Cork, Ireland).

### MR DWI analysis

The conventional ADC value was calculated by fitting the b_0_ image and DWI at each b-value other than 0 s/mm^2^ into the mono-exponential equation (Eq. (1)) [29], where S_b_ is the diffusion weighted signal intensity for the b-value, and S_0_ is the signal intensity obtained with the b_0_ value.

$${\text{S}}_{\text{b}}/{\text{S}}_{0}=\exp\left(-\text{b}\times \text{ADC}\right)$$ (1)

ADC_uh_ were calculated by fitting all diffusion weighted images and b_0_ images into a tri-component model (Eq. (2))[12].

$${\text{S}}_{\text{b}}/{\text{S}}_{0}=\{\begin{array}{c} \text{f}\times \text{exp}\left(-\text{b}\times {\text{D}}^{*}\right)+\left(1+\text{f}\right)\times \exp\left(-\text{b}\times \text{D}\right),\text{b}\leq 1500\;\sec/{\text{mm}}^{2}\\ \exp\left(-\text{b}\times {\text{ADC}}_{\text{uh}}\right),\text{b}\geq 1500\;\sec/{\text{mm}}^{2} \end{array}$$ (2)

where the diffusion weighted signal S_b_ is fit to the bi-exponential equation when b-values are less than or equal to 1,500 sec/mm^2^. ADC_uh_ is the apparent diffusion coefficient calculated by fitting the seven ultra-high b-values (1,500, 2,000, 2500, 3,000, 3,500, 4,000, 4,500 sec/mm^2^) to the mono-exponential equation. D is the true diffusion coefficient that reflects random motion of intra- and intercellular water molecules (slow component of diffusion); f is the fraction of the diffusion linked to microcirculation, and D* is the diffusion parameter representing incoherent microcirculation within the voxel (perfusion-related diffusion, or fast component of diffusion).

The stretched-exponential model is described as follows (Eq. (3))[10]:

$${\text{S}}_{\text{b}}/{\text{S}}_{0}=\exp(-{\left(\text{b}\times \text{DDC}\right)}^{\alpha }$$ (3)

where S_b_ is the signal magnitude with diffusion weighting b, S_0_ is the signal magnitude with no diffusion weighting, the α index relates to intravoxel water diffusion heterogeneity, varying between 0 and 1, and the DDC is the distributed diffusion coefficient, representing mean intravoxel diffusion rates.

### Quantitative image analysis

All data were analyzed and processed on a GE ADW4.6 workstation. All regions of interests (ROIs) were determined by two radiologists (Dr. Y.-C.H. and L.-F.Y., with 12 and 6 years of experience, respectively) at the workstation.

**Figure 5:**
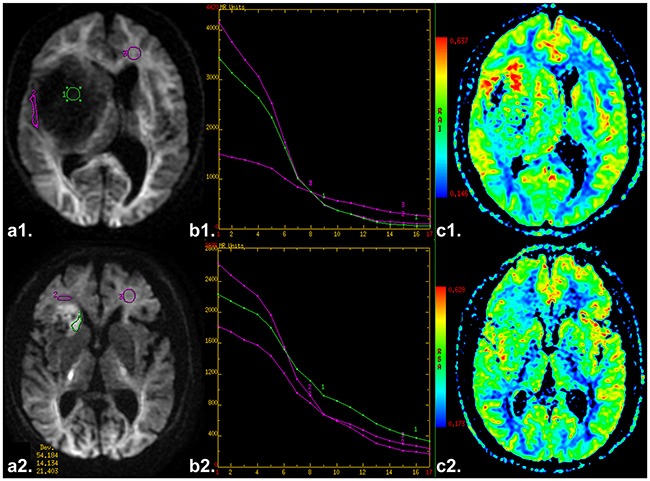
**a1** and **a2**. Axial diffusion-weighted trace image (b=4500 sec/mm^2^) shows ROIs in placed in the solid tumor parts, edema and contralateral healthy white matter area, respectively. **b1** and **b2**. The diffusion signal decay curve over a wide-range of b values (up to 4,500 sec/mm^2^). **c1** and **c2**. Axial ADC_uh_ map. (a1-3. a case of low grade glioma. b1-3. a case of high grade glioma).

First, we reviewed the conventional plain and contrast-enhanced MRI images carefully to determine the solid part of each tumor. Next, the DWI data were analyzed. A freehand region of interest (ROI) was traced using an electronic cursor, which was placed to include the solid elements of tumor by defining ROI based on the relatively higher signal intensity on DW image and lower ADC value in ADC map, avoiding large vessels, hemorrhagic, cystic and necrotic areas. The mean ROI area of the lesions was 94.1 ± 76.0 mm^2^. The standardized ROI were placed in the peritumoral edema and contralateral normal appearing white matter (WM) to calculate the ADC_uh_edema_ and ADC_uh_wm_ value (as shown in Figure 5a1 or 5a2), respectively. The parameter maps of ADC, ADC_uh_ (as shown in Figure 5c1 or 5c2), DDC, α, and tri-exponential diffusion signal decay curve (as shown in Figure 5b1 or 5b2) over a wide-range of b-values (up to 4,500 sec/mm^2^) were automatically generated, and the mean ADC, ADC_uh_, DDC and α values in the ROIs were obtained, respectively.

In addition, estimated signal-to-noise (SNR) was calculated for gray matter (GM), WM and lesions at 4500 sec/mm^2^ b-values as mean SI of all ROIs divided by standard deviation of background noise (measured on a small ROI outside the signal region) [30]. In all subjects, the mean SNR at the highest b-value (4,500 sec/mm^2^) was large enough to ensure proper depiction of the signal, and the mean SNR (range) for WM, GM and lesions were as follows: 70.0 (23.6 - 280.2), 42.8 (17.3 -186.4) and 44.3 (20.0 - 249.5), respectively.

### Pathologic diagnosis

The final diagnosis was determined by surgical findings and confirmed on histopathological examination. Tissue samples obtained from the specimens were routinely processed and stained for hematoxylin and eosin (H&E). Pathologic analysis was performed by the expert in the pathological department (Dr. S.-M.W, with more than 8 years of experience), who was blinded to the clinical and MR findings. Classical histological classification and malignancy grading is based on the criteria of the 2007 World Health Organization (WHO) classification of tumors of the central nervous system [31]. WHO grade II and III, or grade III and IV gliomas were included in the group of high-grade tumors.

### Statistical analysis

Numerical variables were denoted as the mean and standard deviation. The Kolmogorov–Smirnov (K–S) test was used for assessing the normality of data distribution. Parameters of conventional ADC values, ADC_uh_, ADC_uh_edema_, ADC_uh_wm_, DDC and α values were compared for the differences between the low-grade gliomas (LGG: WHO I and II) and high-grade gliomas (HGG: WHO III and IV) by using independent sample t test. Receiver operating characteristic (ROC) analyses were performed to determine optimal thresholds for differentiating the LGG from HGG by conventional ADC, ADC_uh_, DDC and α values, respectively. The diagnostic sensitivity, specificity, and AUC for each parameter were calculated. The ICC were used to assess the data consistency of repeated measures of the same parameter. The ICC was interpreted as poor if it was less than 0.4, as moderate when it was ≥ 0.4 but < 0.75, and as good when it was > 0.75. P < 0.05 indicated a statistically significant difference. All statistical analyses were performed with IBM SPSS 20.0 software (IBM Corp, Chicago, IL, USA).

## CONCLUSION

Different DWI models can be used for accurate preoperative glioma grading. However, the ADC_uh_ value exhibited greater efficacy and reliability than variables from conventional mono-b-value and stretched-exponential models.

This work was supported by the Shaanxi Province Natural Science Foundation (No. 2014JZ2-007).
